# Optimizing an mHealth Intervention to Improve Uptake and Adherence to HIV Pre-exposure Prophylaxis in Young Transgender Women: Protocol for a Multi-Phase Trial

**DOI:** 10.2196/37659

**Published:** 2022-05-19

**Authors:** Karen Kolmodin MacDonell, Bo Wang, Nittaya Phanuphak, Rena Janamnuaysook, Peevara Srimanus, Chokechai Rongkavilit, Sylvie Naar

**Affiliations:** 1 Department of Family Medicine and Public Health Sciences School of Medicine Wayne State University Detroit, MI United States; 2 Department of Population and Quantitative Health Sciences University of Massachusetts Chan Medical School Worcester, MA United States; 3 Institute of HIV Research and Innovation Bangkok Thailand; 4 Center of Excellence in Transgender Health (CETH) Chulalongkorn University Bangkok Thailand; 5 Department of Pediatrics University of California San Francisco-Fresno Branch Campus Fresno, CA United States; 6 Center for Translational Behavioral Research Florida State University Tallahassee, FL United States

**Keywords:** transgender women, PrEP, HIV prevention, mHealth, motivational interviewing, Thailand, mobile phone

## Abstract

**Background:**

Vulnerable adolescents and emerging adults (aged 18-29 years), particularly young transgender women, are among the fastest-growing HIV positive populations worldwide. Thailand has the highest adult HIV seroprevalence in Asia, with a rate of infection among this population of 18%. Widespread technology offers opportunities for innovative mobile health (mHealth) interventions. Pre-exposure prophylaxis (PrEP) is an efficacious HIV prevention strategy recommended for at-risk individuals. PrEP is highly effective when taken as prescribed, but uptake and adherence have been low, with high discontinuation rates among youth.

**Objective:**

We propose to develop and pilot a multi-component, technology-based intervention to promote PrEP usage. We will adapt an existing 2-session, technology-delivered, motivational interviewing-based intervention to focus on PrEP use in transgender women in Thailand. We call this the Motivational Enhancement System for PrEP Uptake and Adherence (MES-PrEP). We will also refine and enhance YaCool, a mobile app with integrated text messaging developed and used clinically by our Thai team. The new version of the app is called Enhanced YaCool, and it enables self-management of gender and sexual health (including PrEP). Our primary aim is to develop and assess the preliminary efficacy of this mHealth intervention.

**Methods:**

We will utilize a multiphase optimization strategy (MOST) to identify the most effective intervention component or combination of components to improve PrEP usage in Thai transgender women. The study includes two phases: phase I (R21) includes qualitative interviews with key stakeholders to explore barriers and facilitators of PrEP usage through thematic analysis to inform intervention adaptation. Following this, we will adapt and beta-test MES-PrEP and Enhanced YaCool for functionality and feasibility using a community advisory board of HIV-negative Thai transgender women. In phase II (R33), we will conduct a MOST design-based trial to evaluate the feasibility, acceptability, and preliminary efficacy of MES-PrEP and Enhanced YaCool. Eighty HIV-negative participants who are currently taking PrEP and 80 participants who are not will be randomized to four conditions: (1) standard PrEP counseling (the control condition); (2) MES-PrEP and standard PrEP counseling; (3) Enhanced YaCool and standard PrEP counseling; and (4) MES-PrEP, Enhanced YaCool, and standard PrEP counseling. Feasibility and acceptability of the intervention will be assessed through usage patterns and the System Usability Scale. Preliminary impact will be assessed by evaluating the proportion of participants who initiate PrEP and their level of adherence to PrEP. Assessments will be at baseline and 1, 3, 6, 9, and 12 months postintervention. Biomarkers of adherence to PrEP, HIV, and other sexually transmitted infections will be collected.

**Results:**

Upon project completion, we will have an optimized mHealth intervention to support the use of PrEP by transgender women that will be ready for testing in a larger efficacy trial.

**Conclusions:**

Even though transgender women in Thailand face increasing risks of HIV, few interventions have targeted them. Effective developmentally and culturally tailored interventions are needed to prevent HIV transmission in this high-risk population.

**Trial Registration:**

ClinicalTrials.gov NCT05262426; https://clinicaltrials.gov/ct2/show/NCT05262426

**International Registered Report Identifier (IRRID):**

PRR1-10.2196/37659

## Introduction

### Background

Transgender women are disproportionately affected by HIV globally [1]. HIV prevalence rates among transgender women are 15% to 28% in many countries [2], and higher in young transgender women [3-5]. Young transgender women (aged 18-29 years) are in emerging adulthood, a period marked by increased independence, identity development, and risk-taking [6,7]. Thailand has the highest adult HIV seroprevalence in Asia (1.1% in 2017) [8]. The rate of new HIV infections among Thai transgender women has been increasing in the past decade [9], with a prevalence rate reaching 18% [10]. Transgender women face stigma related to gender identity, limited employment options, and often engage in high-risk behaviors (eg, sex work and substance use) [11-16]. Interventions designed specifically for the needs of this high-risk population are critical to the success of the United Nations AIDS Fast Track Strategy: ending the AIDS epidemic by 2030 [17]. Widespread technology use offers opportunities for innovative mobile health (mHealth) interventions for young people, including transgender women [18-20].

Pre-exposure prophylaxis (PrEP) is highly effective when taken as prescribed, and daily use has been shown to reduce the risk of HIV infection by 92% [21], but PrEP uptake among transgender women worldwide is low. Studies have shown that this is primarily due to lack of awareness, rather than lack of willingness to use PrEP [22,23]. Further, adherence among transgender women has been found to be only 18%, compared to 52% among men who have sex with men [24], yet PrEP research has primarily focused on men who have sex with men [25]. In the Princess PrEP program, the largest PrEP program in Thailand, 232 transgender women began using PrEP in 2019, but only 43 were using it at month 3, reiterating the challenges of adherence among this population [26]. Common facilitators of PrEP uptake among transgender women include a high perception of HIV risk, accurate knowledge of PrEP, and access to support services [27,28]. Barriers to PrEP uptake and adherence include concerns about side effects, especially interactions with gender-affirming hormone therapy, a low perception of HIV risk, the stigma of HIV, health system inaccessibility, exclusion of transgender women from advertising, and lack of research [27-29], especially in low- and middle-income countries. Effective and sustainable behavior-change interventions are needed to promote PrEP uptake and adherence in high-risk populations like transgender women in Thailand and end the global HIV epidemic.

mHealth may offer a powerful tool to promote behavior change, particularly when based on evidence-based interventions, such as motivational interviewing (MI) [30,31]. Mobile apps that offer feedback and self-monitoring of health have been associated with improved health outcomes [32]. There is also evidence that text message reminders can improve adherence to prevention and treatment across many clinical conditions, especially among emerging adults [18,33]. Text messaging and mobile apps may have great potential to promote health self-management and behavior change in young transgender women, but are currently underutilized [7]. Mobile interventions are useful among emerging adults due to nearly universal technology use [34,35], and appear to increase PrEP adherence among young people at risk for HIV [36,37]. Still, very few technology-based interventions have targeted transgender women.

### Study Aims

In the proposed study, we will develop 2 potentially synergistic, technology-based, theory-driven interventions aimed at maximizing PrEP usage. We propose to develop and pilot a multi-component, technology-based intervention based on the principles of MI to promote PrEP usage. This process will include 2 guiding frameworks: the ADAPT-ITT (assessment, decision, adaptation, production, topical experts, integration, training, testing) model of intervention adaptation, and a multiphase optimization strategy (MOST) design to identify the most effective culturally and developmentally tailored intervention to address PrEP usage in this population. In phase I (R21), we will adapt an existing 2-session, technology-delivered, MI-based intervention to focus on PrEP uptake and adherence in Thai transgender women who are HIV negative through a systematic, multi-step process [38] to develop what we term the Motivational Enhancement System for PrEP Uptake and Adherence (MES-PrEP). MES-PrEP sessions will be completed on participants’ internet-connected mobile devices (eg, smartphones) while at clinics for this study. We will also refine and enhance YaCool, a mobile app with integrated text messaging developed and used clinically by our Thai team. The refined version is called Enhanced YaCool and will enable self-management of gender and sexual health (including PrEP) in transgender women. These intervention components will be integrated to enhance the impact of a 2-session intervention through daily health self-management. In phase II (R33), we will utilize a MOST framework with a factorial design to efficiently identify the most effective component or combination of components to promote PrEP uptake and adherence in young Thai transgender women. This study may pave the way to scaled-up PrEP implementation, a crucial step in eliminating the HIV epidemic among transgender women in Thailand.

### Defining the Theoretical Model of Behavior Change

**Figure 1:**
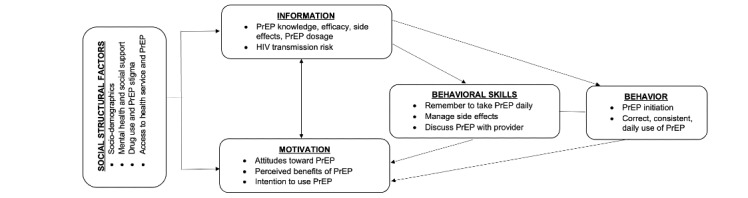
Integrated application of the information-motivation-behavioral skills model and the socioecological model for uptake and adherence. PrEP: pre-exposure prophylaxis.

The information-motivation-behavioral skills (IMB) model and socioecological model (SEM) will serve as conceptual frameworks for the intervention and process of adaptation. Both have been applied to a variety of health promotion topics, including HIV health behaviors [39-41] and PrEP use [42,43]. While IMB includes only individual-level factors, SEM captures individual, structural, and social-level factors. Combining these frameworks may result in a more robust theoretical model. The proposed intervention is primarily focused on behavior change at the individual level, but also addresses structural and social level factors, such as stigma, depression, and access to PrEP. IMB describes behavior change as the result of the joint function of three elements: information about risky behaviors (ie, the risk of not using PrEP) and alternatives (such as using PrEP), the motivation to change behavior, and the behavioral skills to perform the behavior (ie, self-efficacy) (Figure 1). We will develop content to address each of these elements to facilitate PrEP usage among young Thai transgender women.

### The Intervention Components

#### MES-PrEP Intervention

MES-PrEP is a brief, tailored, 2-session intervention deliverable to any internet-enabled device via the Computerized Intervention Authoring System (version 3.0). A 3D avatar speaks, moves, and displays emotions. At the start of each session, the participant chooses an avatar, who delivers the intervention in a way that closely follows the most recent edition of MI (MI-3) [44]. The avatar evokes importance and confidence (key components of motivation) with MI strategies such as identifying pros of behavior change, providing affirmations to reinforce “change talk” and boost confidence, and identifying strengths and resources. MI strategies are utilized within the motivational enhancement system to promote autonomy, boost self-efficacy, and identify social supports, while also addressing stigma and coping. The intervention manages “counter-change talk” by having the avatar reflect without judgment and provide statements to emphasize autonomy. As with interaction with a human counselor, interactions are synchronous and not reliant on feedback at the completion of the session. Small amounts of education about PrEP are integrated with motivational elements. The intervention is branched, with participants assigned to different branches based on their rating of importance of PrEP and their confidence in taking it; each branch has content tailored to these responses. For example, participants who give a low rating to the importance of PrEP are assigned to a branch that focuses on decisional balance exercises, while those who give a high rating for importance are assigned to a branch that provides reinforcement. The participants are placed in branches that provide content exploring stigma, autonomy, self-efficacy, and social supports. Participants are given feedback on their PrEP knowledge and information on the protective effects of PrEP adherence. Each participant is then asked to set a goal: obtain a PrEP prescription, maintain optimal adherence, practice steps, or think about it more, and asked to identify possible barriers to usage (such as stigma, forgetting, or side effects) and form plans for overcoming these barriers. In the second session (1 month later), participants are assigned to new branches based on whether they met their goal (this branch provides reinforcement and encourages planning for continued success), partially met their goal (this branch focuses on identifying plans for overcoming barriers), or did not meet their goal (this branch reviews the importance of PrEP and encourages confidence in it). The participants may choose a new goal or continue the same goal.

#### YaCool

YaCool is a mobile app developed by our Thai collaborators to support self-management of gender and sexual health in transgender women. Self-management of health requires skills such as problem-solving, goal-setting, and action planning [45], as well as health behaviors required to effectively remain healthy (eg, adherence to prescriptions and keeping appointments). Self-management assumes autonomy and that emerging adults will increase their responsibility for their own health decisions. YaCool is a passcode-protected app that is installed on users’ smartphones and allows users to personalize reminders to take PrEP, record taking PrEP, and calculate adherence. YaCool includes a diary to record sexual activity, the use of preventive methods, and substance use. It is set up to provide personalized recommendations for PrEP, confirmed by clinic health care staff. YaCool also offers personalized appointment reminders and lab results (eg, testing for sexually transmitted infections [STIs]). We will further refine the content in YaCool and its appearance to allow more seamless integration with MES-PrEP, and will add new features from a list generated during formative work. Our Thai team conducted a focus group with 7 young transgender women. The focus group recommended new features to enhance the YaCool app: (1) lab results to track hormone levels; (2) self-assessment tools (eg, for depression screening); (3) incentives for data input or attendance at clinics; and (4) linkage to mental health providers. In the proposed project, YaCool will be refined and beta tested to develop a new version called Enhanced YaCool to allow transgender women to self-manage gender and sexual health between and after MES-PrEP sessions.

#### Standard PrEP Counseling (Control Condition)

All study participants will receive one-on-one, face to face counseling focused on sexual and behavioral risk assessment for HIV and other STIs and risk reduction. For those who are at high risk but are not yet ready to start PrEP, the focus will be on risk assessment and perception, awareness of PrEP and postexposure prophylaxis, facilitators of PrEP use, and barriers to access to PrEP. For those on PrEP, the focus will be on adherence. Counseling is standard practice within our partner clinics and will last about 15 to 20 minutes.

### Guiding Frameworks

#### The ADAPT-ITT Model

Development and testing of the interventions (MES-PrEP and YaCool) will be guided by the ADAPT-ITT model [46] for both the R21 and R33 phases. This framework is designed to guide adaptation of evidence-based interventions for people with HIV or at risk of contracting it. It balances rigor with pragmatic concerns (such as cost and time) and has been used, including in our previous work, for interventions targeting diverse populations [38]. ADAPT-ITT begins with a needs assessment and progresses to evaluating the efficacy of the intervention. This is appropriate for this proposal because the need to improve PrEP usage in young Thai transgender women has been established, but specific barriers and facilitators are less clear.

#### MOST Design

MOST is an efficient, cost-effective, engineering-based approach to identify effective components at the outset of the intervention development process [47,48]. MOST is able to identify active components, in other words, those that make significant contributions to the overall effect. This analytic approach employs factorial designs [49] to identify key combinations for the final phase (confirmation). This study uses MOST to examine the individual and combined effects of MES-PrEP and YaCool on PrEP usage by transgender women.

## Methods

### Study Setting

Transgender women will be recruited from 2 large clinics in Bangkok, Thailand, for both study phases. Tangerine Community Health Clinic is the first transgender-specific sexual health clinic in Asia and offers gender-affirming, comprehensive, integrated health care. Since 2015, over 3600 transgender clients have received HIV counseling and testing by trained transgender and gender-sensitive health care staff. Rainbow Sky Association of Thailand Clinic is among the first community-based organizations to serve at-risk populations in Thailand. Through funding support by the US Agency for International Development/The US President’s Emergency Plan for AIDS Relief and a partnership with the Institute of HIV Research and Innovation, this clinic has implemented the Key Population-Led Health Services Model since 2015. The clinic is located in a densely populated area in Bangkok and provides HIV-related health services for key populations, including transgender women.

### Study Design

There are two phases in the study: phase I (R21) and phase II (R33). 

#### Phase IA and Phase IB: Formative Research and Intervention Adaptation (R21)

In phase IA, we will conduct interviews with 20 HIV-negative transgender women (including 10 who are naive to PrEP and 10 who are currently on PrEP; the latter group will include 5 participants with good adherence) and 10 health care providers or community health workers from our partner clinics to explore factors related to PrEP uptake and adherence and guide adaptation of our intervention frameworks to the target population. An interview guide will be created by the investigator team. Based on the IMB model, we will explore three key aspects: (1) information, which explores transgender women’s knowledge of PrEP and HIV transmission risk, interest in using PrEP, and the perceived advantages and disadvantages of PrEP; (2) motivation, which includes their attitudes toward PrEP, perception of HIV risk, and reasons for accepting or declining PrEP; and (3) behavioral skills, which examines specific behavioral skills relevant to PrEP initiation and adherence, facilitators and barriers to PrEP initiation and adherence, reasons for keeping scheduled appointments or filling PrEP prescriptions, and reasons for discontinuing or restarting PrEP. We will also explore sources of social support and past successes, as well as strategies and solutions to overcoming barriers. Health care providers will be asked about barriers and facilitators of PrEP and experiences with clients and will be prompted to identify intervention topics related to PrEP. The responses will be used to broadly inform intervention content (eg, what modules to include in the intervention framework), but also more specifically (eg, response options for barriers and sources of social support). We will also prompt the participants for feedback on the content of the intervention (such as by asking them what they would like to know more about PrEP) and delivery (such as by asking how often they would like to receive reminders to take PrEP and what components they would add to YaCool). Interviews will be audiotaped, transcribed, and translated into English by an independent translator. Interviews will last about 30 minutes. The transgender women and health provider participants will receive $25 for their time.

In phase IB, we will take a motivational enhancement intervention framework that was developed for youth living with HIV in the United States and utilize our results to adapt it to our target population. We will also add features and enhancements to YaCool. The adaptation process will involve two steps: (1) initial programming of the adapted sessions and (2) evaluation of the cultural and developmental acceptability of the intervention content via a community advisory board (CAB) and beta testing by 10 HIV-negative young transgender women. The transgender women will complete 2 MES-PrEP sessions over 1 month. Each session will be videotaped. The transgender women will also download Enhanced YaCool for daily use between and after PrEP sessions. We will administer assessments during beta testing and review the content for cultural and developmental relevancy and MI consistency. During this period, staff will call the participants to check for issues with YaCool. A brief interview will be conducted after beta-testing to (1) identify any technical or other issues with either component, and (2) solicit input on how to improve the delivery and content. Throughout phase IB, we will conduct troubleshooting and revise the programming. Adaptation, production, and refinement will result in the final versions of MES-PrEP and Enhanced YaCool being developed by the end of the R21 phase.

#### Selection of CAB

CAB members (N=8-10) will be randomly selected from transgender women who complete interviews. Members will meet quarterly (with additional meetings as needed). The primary role of the CAB will be to provide iterative feedback on study materials, recruitment and retention strategies, intervention approaches and content, and disseminating study findings. Operational guidelines will follow best practices for community-participatory research (such as establishing the CAB’s function and promoting empowerment). [50] Any inconsistencies in the feedback will be resolved during the meeting, or if necessary, via discussions with the Thai-US team. Detailed notes will be taken during meetings. Study materials will be revised based on CAB feedback. CAB members will receive $25 per meeting.

#### Phase II: Pilot MOST Design-Based Trial (R33)

We will launch a pilot trial using a full factorial design to evaluate the feasibility, acceptability, and preliminary efficacy of intervention components to increase PrEP uptake and adherence among young Thai transgender women. The trial will examine (1) the effect of each component on participants who are currently on PrEP but not adherent and participants who have not started PrEP and (2) whether the presence or absence of a component has an impact on the performance of other components. While there exist several approaches to construct a MOST trial, we will use a full factorial design given its relative simplicity and cost efficiency [48,51]. For our MOST trial, the 2 intervention components each have 2 levels, resulting in a 2^1^2^1^ full factorial with 4 experimental conditions. MOST requires a well-defined optimization criterion. We will use PrEP uptake and adherence as the measure of success. In order to build an optimized intervention for participants who are on PrEP and participants who are not on PrEP, we will enroll 80 participants who are on PrEP and 80 participants who have not started PrEP. The 80 participants who have not started PrEP will all be individuals who are at high risk and are eligible for PrEP but have not yet decided to start PrEP.

Standard PrEP counseling will be provided to all participants at the beginning of the trial and treated as a constant component in the experiment. After counseling, participants will be randomly assigned to 1 of the 4 experimental conditions (Table 1). Separate randomization for participants who are on PrEP and participants who have not started PrEP will be conducted via Qualtrics (Qualtrics XM). The primary outcome will be PrEP uptake and adherence assessed through computer-assisted self-interviewing (CASI) and dried blood spot (DBS) testing. The main effects of the intervention components and the interactions between them will be estimated using ANOVA. The goal of the R33 phase is to examine intervention strategies based on preliminary effect sizes, feasibility, and acceptability and prepare for a fully powered randomized controlled trial to evaluate the optimized intervention package.

**Table 1:** Study experimental conditions.

Experimental condition (n=20 for each condition)	Standard PrEP^a^ counseling	Motivational enhancement system for PrEP	Enhanced YaCool
1	Yes	Yes	Yes
2	Yes	Yes	No
3	Yes	No	Yes
4	Yes	No	No

^a^PrEP: pre-exposure prophylaxis.

### Study Sample

Eligibility criteria, screening, and consent are the same for the R21 and R33 phases. All participants will be transgender women who are eligible based upon inclusion criteria consistent with US Centers for Disease Control and Prevention and Thailand Ministry of Public Health PrEP guidelines [52,53]: (1) age 18 to 29 years; (2) male sex at birth; (3) self-identification as a woman or transgender woman or cultural identification with the female spectrum; (4) laboratory-confirmed HIV negative status; (4) self-reported recent history of condomless sex; (5) ability to understand, read, and speak Thai; and (6) either no existing use of PrEP (group 1, PrEP naive) or currently on PrEP but not adherent (taking ≤3 pills/week) in the past month (group 2, PrEP users). The exclusion criteria are (1) a serious cognitive or psychiatric problem compromising the ability to provide informed consent; (2) active suicidal ideation or major mental illness (eg, untreated psychosis or mania) at the time of the interview (these patients will be referred for treatment); (3) laboratory or clinical findings that would preclude PrEP initiation (eg, decreased creatinine clearance); and (4) current enrollment in another HIV intervention study. We will recruit transgender women from a broad range of socioeconomic backgrounds; therefore, mobile phone ownership is not required for participation, and a mobile phone will be provided at no cost to participants who do not have one. 

### Sample Size of Trial

Using G*power 3.1.9 (Heinrich-Heine-Universität Düsseldorf) for the ANOVA, we will set the effect size *F* at 0.35, α at .05 and the sample size at 80 (20 for each experimental condition). The calculated statistical power is 80% for detecting the 2 main effects, MES-PrEP and YaCool, and 73% for detecting their interaction. The pilot trial is not an efficacy study, and therefore is not fully powered to detect both main effects and interactions, but has a sample large enough to detect a medium to large effect size for MES-PrEP and YaCool.

### Recruitment and Enrollment Procedures

Recruitment will be led by a Thai transgender woman researcher with 5 years of experience developing materials and recruiting young transgender women for HIV prevention research. Materials will be placed at physical and online venues, including study clinical sites and community venues that serve transgender women (eg, health centers, bars and clubs, and community organizations). Participants will also be recruited by paid study recruiters who identify as transgender women using direct outreach in the community. These efforts have been successful in other projects seeking to enroll transgender women [26,54,55], and as such, we do not anticipate having problems reaching our target sample.

Potential participants will complete a prescreener over the phone or in person. If eligible and interested in participating, the transgender women will complete an in-person screening at one of the study clinics. After providing verbal consent, they will complete a prebaseline survey assessing basic demographic information, history of HIV and STIs, HIV testing, access to HIV and STI diagnostic and treatment services, recent sexual behavior, use of PrEP, and knowledge and acceptance of PrEP. They will undergo HIV testing (with a fourth-generation serum) and screening for hepatitis B (with surface antigens and antibodies), hepatitis C (with antibodies), and renal insufficiency (with serum creatinine testing) to confirm their eligibility for PrEP and the study. Research staff will determine if the transgender women meet the inclusion criteria and have completed informed consent. The screening visit, enrollment, and baseline assessment should occur on the same day, or failing that, within one week. After signed consent is obtained, participants will be asked to provide contact information for a family member or friend who can be called if they cannot be reached.

### Data Collection Tools and Procedures

Self-report measures will be administered using the cloud-based CASI system in Thai at baseline, immediately postintervention at the end of the first month, and at 3, 6, 9 and 12 months. In CASI, the participant sees each question and a list of responses on a private iPad screen. The response is entered by pressing a number keypad. The data are stored directly in the password-protected Qualtrics system. CASI elicits more valid and reliable data than face-to-face or written questionnaires [56-58]. The topics we assess are sensitive; CASI allows greater privacy for honest responses [59,60] and reduces missing data and interviewer bias. Our team has been using Qualtrics for CASI for over 8 years.

The primary acceptability outcome will be measured by the System Usability Scale, a 10-item Likert scale giving an overall view of subjective assessments of usability. The System Usability Scale is technology agnostic and provides a global measure of system satisfaction [61]. A score of >50 (out of 100) indicates that an intervention is acceptable [62]. We will also assess interest in future use at study completion. For feasibility, we will assess the number of responses and frequency of usage of Enhanced YaCool, the number of MES-PrEP sessions completed, cumulative time spent in MES-PrEP, and recruitment rates. PrEP uptake will be assessed using self-reported measures (from participants who left the clinic with an emtricitabine/tenofovir disoproxil fumarate [TDF/FTC] prescription), confirmed via chart review and pharmacy records. Adherence to PrEP will be measured using (1) a young adult adherence interview with a visual analog scale [63]; (2) a 4-week percentage taken measure (answering the question “What percentage of the time were you able to take all your PrEP medications in the past 4 weeks?”) [64]; and (3) biological testing using a DBS report on 80% TDF/FTC adherence after at least 3 weeks of regular adherence. The results of DBS testing will be triangulated with the self-reports. We will assess IMB model constructs—knowledge, attitudes, motivations, and behavioral skills related to taking PrEP, and important contextual factors (age, education, depression, and PrEP-related stigma).

### Data Analysis

#### Intervention Evaluation

We will estimate retention rates, overall and per condition, and examine differences across conditions using the chi-square test. We will assess differences in acceptability using the *t* test or the Mann-Whitney test. The minimum criteria for acceptability and feasibility will be point estimate for mean System Usability Scale score ≥50 and having at least 50% of participants respond to YaCool at least once and complete at least one MES-PrEP session [62]. Using a 2^1^2^1^ full factorial design, we will test the main effects and interactions between components with an ANOVA using effect coding (1 = “no” condition; +1 = “yes” condition) rather than dummy coding (ie, 0, 1). A participant will be classified as adherent if her tenofovir level in DBS reflects the use of TDF/FTC tablets 4 to 7 days a week over the prior 6 weeks (a level with an almost 100% preventive effect). Participants whose tenofovir levels cannot be assessed will be classified as nonadherent. The intervention effect on PrEP uptake and adherence will be further assessed using mixed-effects modeling controlling for potential confounding factors (eg, age and education) and possible baseline differences. Missing data will be handled using full information maximum likelihood estimation. Effect sizes will be estimated for the primary outcomes. All analyses will be conducted using the SAS 9.4 statistical software package (SAS Institute Inc).

Structural equation modeling will be used to examine the extent to which the targeted variables (eg, information, motivation, and self-efficacy) mediate the intervention effect. Guided by our conceptual model, we will assess the effects of intervention on the mediator and the effects of the mediator on the outcome, controlling for other mediators and confounders, and we will then estimate the proportion of the intervention effect explained. Mediation models will be tested using MPlus (Muthén & Muthén). Given the limited sample size, conclusions from the mediation analyses will be limited and treated with caution, but modeling will allow us to explore the constructs of IMB and potential predictors in preparation for a larger efficacy trial.

#### Component Selection

We will make a preliminary selection of components that have achieved main effects (ie, that exceed statistical significance or have a medium to large effect size). This selection will be reevaluated if we find any substantial interaction effects and understand how the components work in combination. Optimization criteria (ie, PrEP uptake and adherence) will then be combined with other information (eg, cost, feasibility, and scalability) to make the final component selection [65]. This information will guide assembly of an optimized intervention package that achieves the target outcomes with the least resource consumption and participant burden.

### Ethics Approval

The research protocol and methodology were reviewed and approved by the by the University of Massachusetts Chan Medical School Human Investigation Committee and the Research Ethics Review Committee of Chulalongkorn University in Thailand (H00023527). The trial has been registered at Clinicaltrials.gov (NCT05262426).

## Results

Recruitment for this study began December 2021 for phase I. Qualitative interviews were completed with 28 transgender women in February, 2022. Analyses are ongoing. Results will be disseminated to key stakeholders and used to inform intervention adaptation and refinement. Phase II, the pilot feasibility and acceptability trial, will begin in August 2023 following approval from the National Institutes of Health to begin the next phase. The findings will be disseminated to stakeholders and communities of interest using peer-reviewed journals, academic conferences, and other communication channels.

### Transition Milestones

The major goal of the R21 phase is to develop MES-PrEP and Enhanced YaCool and to establish the initial feasibility of this mHealth intervention. Formative research will inform development and refinement, followed by adaptation and production of the mHealth intervention. The final step of ADAPT-ITT is testing, which will include a small pilot feasibility testing study with 10 participants (the R21 phase), and a randomized controlled trial of the mHealth intervention (the R33 phase). We will also develop optimized research protocols for a large efficacy trial.

## Discussion

### Principal Aims

The main aim of this study is to develop an optimized, culturally tailored, mobile intervention to improve PrEP uptake and adherence among transgender women in Thailand. Transgender women are among the most at-risk groups for HIV transmission, yet PrEP usage has remained low. We propose to adapt 2 existing intervention components for this target population using input from key stakeholders within our participant population. Using an mHealth approach, we will test different combinations of our intervention components to assess which combination results in the best outcomes in terms of feasibility, acceptability, and signals of intervention efficacy. Based on our theoretical models of behavior change (IMB and SEM), we anticipate that the combination of all 3 intervention components (MES-PrEP, YaCool, and standard PrEP counseling) will be found to be the optimal intervention condition, but this is yet to be determined. If successful, this study will be among the first to identify an optimized intervention package for transgender women in Thailand toward improving PrEP usage and ending the HIV epidemic globally. It may also be more sustainable than traditional in-person approaches to behavioral intervention because of the use of relatively low-cost and widely available mobile technologies.

### Planned Next Steps

The project will be conducted to develop an optimized technology-based intervention for HIV-negative young Thai transgender women and prepare for a future large-scale randomized controlled trial. For a fully-powered R01 project, we propose a multisite study testing the efficacy of mobile MES-PrEP and Enhanced YaCool using a type 2 effectiveness-implementation design [66] with testing of intervention effectiveness in eliciting the desired outcomes across the PrEP cascade and gathering of information on the implementation of technology-based intervention, including cost analysis to assess the relative cost of implementing the intervention.

### Limitations

Retention and differential loss to follow up are threats to internal validity. Possible barriers are no-shows at clinic visits or refusal to participate. Techniques we will use to increase compliance with appointments include advanced scheduling, reminders between visits and prior to follow-up surveys, texts, emails, and compensation for time spent. If a participant misses a follow-up survey or intervention session, additional outreach will be made to support engagement. Threats to internal validity of the study may also arise if there is insufficient attention to quality assurance during data collection and intervention delivery. Possible technological difficulties with the software, text messaging, and server are another concern. However, we have experience across multiple projects using these technologies and troubleshooting and tracking any issues, and technical assistance and software programming are written into the budget.

### Conclusions

This study addresses a critical problem (high HIV incidence and low PrEP use) among an at-risk population (transgender women). We are developing 2 potentially synergistic, technology-based, theory-driven interventions aimed at maximizing PrEP usage. Thailand was part of the world’s first PrEP trial (iPrEP) and several PrEP demonstration projects, yet uptake and adherence remain low. Our study is timely and relevant as it addresses major challenges related to PrEP use. It is responsive to the US Preventive Services Task Force’s recommendations calling on research to increase PrEP uptake and adherence [67]. This study may pave the way to scaled-up PrEP implementation, a crucial step in eliminating the HIV epidemic among transgender women in Thailand.

## Abbreviations

ADAPT-ITT: assessment, decision, adaptation, production, topical experts, integration, training, testing
IMB: information-motivation-behavioral skills model
MES-PrEP: Motivational Enhancement System for Pre-Exposure Prophylaxis
MI: motivational interviewing
MOST: multiphase optimization strategy
PrEP: pre-exposure prophylaxis
SEM: socioecological model
STI: sexually transmitted infection
